# Supplementary material for: Midgut Bacterial Microbiota of 12 Fish Species from a Marine Protected Area in the Aegean Sea (Greece)
Source: Microb Ecol. 2022 Dec 19;86(2):1405–15. doi: 10.1007/s00248-022-02154-x (PMC10335961; doi:10.1007/s00248-022-02154-x)
Supplement: Supplementary file 2 — Supplementary file2 (DOCX 1519 KB) [file 248_2022_2154_MOESM2_ESM.docx]

Midgut bacterial microbiota of 12 fish species from a marine protected area in the Aegean Sea (Greece)

Konstantinos Kormas^1*^, Eleni Nikouli^1^, Vasiliki Kousteni^2,3^, Dimitrios Damalas^2^

^1^ Department of Ichthyology and Aquatic Environment, University of Thessaly, 384 46 Volos, Greece

^2^ Institute of Marine Biological Resources and Inland Waters, Hellenic Centre for Marine Research, 710 03 Heraklion, Greece

^3^ Fisheries Research Institute, Hellenic Agricultural Organization Demeter, 640 07 Nea Peramos, Greece

***** Correspondence: [kkormas@uth.gr](mailto:kkormas@uth.gr); Tel.: +30-242-109-3082

**Supplementary file**

**Table S1.** Sampling locations off Gyaros Island, Aegean Sea, Greece.

| **Station ID** | **Location** | **Coordinates** | **Depth of fishing (m)** | **Dominant substrate type** |
| --- | --- | --- | --- | --- |
| St. 1 | Fyllada | 37° 35.421’ N  24° 42.129’ E | 18 | *Posidonia*/Rocky |
| St. 2 | Glaronissi | 37° 34.860’ N  24° 45.015’ E | 17 | *Posidonia*/Rocky |
| St. 3 | Fournaki | 37° 37.486’ N  24° 45.117’ E | 98 | Maerl |
| St. 4 | Colata | 37° 37.323’ N  24° 40.998’ E | 88 | Maerl |
| St. 5 | Fouis | 37° 36.367’ N  24° 38.612’ E | 47 | Rocky |

**Table S2**. Standard biometric measurements of the fish specimens used in the study with the corresponding metadata.

| **Family** | **Species** | **Sampling information** | | | | | |
| --- | --- | --- | --- | --- | --- | --- | --- |
|  |  | **MONTH** | **YEAR** | **STATION** | **MATURITY STAGE** | **TL (mm)** | **M_E_ (g)** |
| Sparidae | *Diplodus annularis* | JUL. | 2018 | 2 | 5 | 163 | 68 |
|  |  | SEP. | 2018 | 2 | 3 | 156 | 66 |
|  |  | FEB. | 2019 | 2 | 3 | 171 | 29.6 |
|  |  | JUL. | 2018 | 2 | 3 | 152 | 52 |
|  | *Diplodus vulgaris* | JUL. | 2018 | 1 | 2 | 182 | 80 |
|  |  | JUL. | 2018 | 1 | 2 | 167 | 53 |
|  |  | JUL. | 2018 | 5 | 2 | 193 | 106 |
|  |  | JUL. | 2018 | 2 | 2 | 202 | 121 |
|  | *Pagrus pagrus* | FEB. | 2019 | 3 | NA | 312 | 409 |
|  |  | FEB. | 2019 | 3 | 5 | 351 | 357.4 |
|  |  | FEB. | 2019 | 3 | 2 | 249 | 222 |
|  | *Pagellus erythrinus* | JUL. | 2018 | 4 | 4 | 306 | 339 |
|  |  | FEB. | 2019 | 3 | 2 | 265 | 226.3 |
|  |  | FEB. | 2019 | 4 | 2 | 360 | 447.9 |
|  | *Spondyliosoma cantharus* | JUL. | 2018 | 5 | 2 | 225 | 193 |
|  |  | JUL. | 2018 | 1 | 2 | 212 | 124 |
|  |  | JUL. | 2018 | 5 | 2 | 221 | 157 |
|  |  | JUL. | 2018 | 5 | 2 | 223 | 161 |
| Scorpaenidae | *Scorpaena porcus* | SEP. | 2018 | 2 | 2 | 188 | 129 |
|  |  | SEP. | 2018 | 1 | 2 | 208 | 165 |
|  |  | SEP. | 2018 | 2 | 2 | 191 | 146 |
|  |  | SEP. | 2018 | 2 | 2 | 194 | 139 |
|  | *Scorpaena scrofa* | JUL. | 2018 | 2 | 2 | 184 | 105 |
|  |  | JUL. | 2018 | 2 | 2 | 207 | 129 |
|  |  | JUL. | 2018 | 2 | 2 | 310 | 514 |
|  |  | JUL. | 2018 | 2 | 2 | 278 | 350 |
| Mullidae | *Mullus surmuletus* | FEB. | 2019 | 4 | 4 | 270 | 263.2 |
|  |  | FEB. | 2019 | 1 | 4 | 255 | 208 |
|  |  | FEB. | 2019 | 3 | 4 | 298 | 314.6 |
|  |  | FEB. | 2019 | 3 | 4 | 290 | 290.4 |
| Scyliorhinidae | *Scyliorhinus canicula* | JUL. | 2018 | 3 | 4 | 409 | 158 |
|  |  | JUL. | 2018 | 3 | 4 | 447 | 435 |
|  |  | JUL. | 2018 | 4 | 4 | 432 | 191 |
|  |  | JUL. | 2018 | 3 | 4 | 422 | 187 |
| Scaridae | *Sparisoma cretense* | JUL. | 2018 | 2 | 5 | 232 | 180 |
|  |  | JUL. | 2018 | 2 | 5 | 278 | 319 |
|  |  | JUL. | 2018 | 2 | 5 | 276 | 317 |
|  |  | JUL. | 2018 | 2 | 5 | 345 | 591 |
| Scombridae | *Euthynnus alletteratus* | FEB. | 2019 | 1 | 2 | 469 | 1195.4 |
|  |  | FEB. | 2019 | 1 | 1 | 392 | 631.8 |
|  |  | FEB. | 2019 | 1 | 2 | 390 | 716.6 |
|  |  | FEB. | 2019 | 1 | 2 | 461 | 1173.8 |
| Uranoscopidae | *Uranoscopus scaber* | SEP. | 2018 | 1 | 1 | 231 | 184 |
|  |  | SEP. | 2018 | 2 | 1 | 275 | 360 |
|  |  | FEB. | 2019 | 2 | 1 | 264 | 306.8 |

**Table S3**. Core (shaded) and most abundant (cumulative relative abundance ≥70% per sample) bacterial operational taxonomic units (OTU) in the midgut of 12 fish species from the Aegean Sea. Greece.

| ***OTU-s*** | ***Phylum*** | ***Class*** | ***Order*** | ***Family*** | ***Genus*** |
| --- | --- | --- | --- | --- | --- |
| OTU-001 | Proteobacteria | Alphaproteobacteria | Rhizobiales | Xanthobacteraceae | *Bradyrhizobium* |
| OTU-002 | Proteobacteria | Gammaproteobacteria | Burkholderiales | Comamonadaceae | *Diaphorobacter* |
| OTU-003 | Proteobacteria | Gammaproteobacteria | Enterobacterales | Pseudoalteromonadaceae | *Pseudoalteromonas* |
| OTU-004 | Proteobacteria | Gammaproteobacteria | Burkholderiales | Comamonadaceae | *Pelomonas* |
| OTU-005 | Firmicutes | Clostridia | Peptostreptococcales-Tissierellales | Peptostreptococcaceae | *Romboutsia* |
| OTU-006 | Firmicutes | Clostridia | Clostridiales | Clostridiaceae | *Clostridium* sensu stricto 1 |
| OTU-007 | Firmicutes | Bacilli | Staphylococcales | Staphylococcaceae | *Staphylococcus* |
| OTU-008 | Bacteroidota | Bacteroidia | Flavobacteriales | Weeksellaceae | *Cloacibacterium* |
| OTU-009 | Proteobacteria | Gammaproteobacteria | Enterobacterales | Vibrionaceae | Unclassified |
| OTU-010 | Proteobacteria | Gammaproteobacteria | Pseudomonadales | Microbulbiferaceae | *Microbulbifer* |
| OTU-011 | Actinobacteriota | Actinobacteria | Propionibacteriales | Propionibacteriaceae | *Cutibacterium* |
| OTU-012 | Firmicutes | Clostridia | Clostridiales | Clostridiaceae | *Clostridium* sensu stricto 1 |
| OTU-013 | Proteobacteria | Gammaproteobacteria | Enterobacterales | Pseudoalteromonadaceae | *Pseudoalteromonas* |
| OTU-014 | Bacteroidota | Bacteroidia | Flavobacteriales | Flavobacteriaceae | *Capnocytophaga* |
| OTU-015 | Firmicutes | Bacilli | Lactobacillales | Streptococcaceae | *Streptococcus* |
| OTU-016 | Firmicutes | Clostridia | Clostridiales | Clostridiaceae | Unclassified |
| OTU-017 | Proteobacteria | Gammaproteobacteria | Enterobacterales | Vibrionaceae | *Thaumasiovibrio* |
| OTU-018 | Firmicutes | Bacilli | Thermicanales | Thermicanaceae | *Thermicanus* |
| OTU-019 | Firmicutes | Bacilli | Mycoplasmatales | Mycoplasmataceae | *Mycoplasma* |
| OTU-020 | Proteobacteria | Alphaproteobacteria | Rhizobiales | Beijerinckiaceae | *Bosea* |
| OTU-021 | Firmicutes | Bacilli | Bacillales | Bacillaceae | *Aeribacillus* |
| OTU-022 | Fusobacteriota | Fusobacteriia | Fusobacteriales | Fusobacteriaceae | *Cetobacterium* |
| OTU-023 | Proteobacteria | Gammaproteobacteria | Xanthomonadales | Rhodanobacteraceae | *Luteibacter* |
| OTU-024 | Firmicutes | Bacilli | Mycoplasmatales | Mycoplasmataceae | *Mycoplasma* |
| OTU-025 | Bacteroidota | Bacteroidia | Flavobacteriales | Flavobacteriaceae | *Polaribacter* |
| OTU-026 | Proteobacteria | Alphaproteobacteria | Rhodobacterales | Rhodobacteraceae | *Ruegeria* |
| OTU-027 | Bacteroidota | Bacteroidia | Chitinophagales | Chitinophagaceae | *Puia* |
| OTU-028 | Proteobacteria | Alphaproteobacteria | Rhodobacterales | Rhodobacteraceae | *Paracoccus* |
| OTU-029 | Patescibacteria | Parcubacteria | *Candidatus* Kaiserbacteria | *Candidatus* Kaiserbacteria | Unclassified |
| OTU-030 | Firmicutes | Bacilli | Bacillales | Bacillaceae | *Anoxybacillus* |
| OTU-031 | Actinobacteriota | Actinobacteria | Micrococcales | Micrococcales *incertae* *sedis* | *Timonella* |
| OTU-032 | Proteobacteria | Gammaproteobacteria | Enterobacterales | Vibrionaceae | *Photobacterium* |
| OTU-033 | Actinobacteriota | Actinobacteria | Micrococcales | Bogoriellaceae | *Georgenia* |
| OTU-034 | Dependentiae | Babeliae | Babeliales | Vermiphilaceae | Unclassified |
| OTU-035 | Proteobacteria | Alphaproteobacteria | Sphingomonadales | Sphingomonadaceae | *Sphingomonas* |
| OTU-036 | Firmicutes | Bacilli | Lactobacillales | Enterococcaceae | *Enterococcus* |
| OTU-038 | Firmicutes | Clostridia | Oscillospirales | Ruminococcaceae | Unclassified |
| OTU-039 | Proteobacteria | Alphaproteobacteria | Rhodobacterales | Rhodobacteraceae | *Paracoccus* |
| OTU-040 | Proteobacteria | Alphaproteobacteria | Azospirillales | Azospirillaceae | *Skermanella* |
| OTU-041 | Proteobacteria | Alphaproteobacteria | Azospirillales | Azospirillaceae | *Skermanella* |
| OTU-042 | Desulfobacterota | Desulfovibrionia | Desulfovibrionales | Desulfovibrionaceae | *Desulfovibrio* |
| OTU-044 | Planctomycetota | Planctomycetes | Pirellulales | Pirellulaceae | *Blastopirellula* |
| OTU-045 | Proteobacteria | Gammaproteobacteria | Pseudomonadales | Cellvibrionaceae | *Cellvibrio* |
| OTU-046 | Firmicutes | Clostridia | Clostridiales | Clostridiaceae | *Clostridium* sensu stricto 1 |
| OTU-047 | Firmicutes | Bacilli | Bacillales | Bacillaceae | *Geobacillus* |
| OTU-048 | Firmicutes | Bacilli | Lactobacillales | Lactobacillaceae | *Lactobacillus* |
| OTU-049 | Firmicutes | Bacilli | Lactobacillales | Streptococcaceae | *Streptococcus* |
| OTU-051 | Bacteroidota | Bacteroidia | Flavobacteriales | Flavobacteriaceae | *Flavobacterium* |
| OTU-053 | Proteobacteria | Alphaproteobacteria | Sphingomonadales | Sphingomonadaceae | Unclassified |
| OTU-054 | Bacteroidota | Bacteroidia | Cytophagales | Hymenobacteraceae | *Rufibacter* |
| OTU-056 | Proteobacteria | Gammaproteobacteria | Enterobacterales | Colwelliaceae | *Colwellia* |
| OTU-058 | Actinobacteriota | Actinobacteria | Micrococcales | Dermacoccaceae | *Kytococcus* |
| OTU-059 | Firmicutes | Bacilli | Lactobacillales | Catellicoccaceae | *Catellicoccus* |
| OTU-060 | Proteobacteria | Alphaproteobacteria | Rhodobacterales | Rhodobacteraceae | *Sulfitobacter* |
| OTU-061 | Proteobacteria | Alphaproteobacteria | Rhizobiales | Beijerinckiaceae | *Methylobacterium-Methylorubrum* |
| OTU-062 | Firmicutes | Bacilli | Erysipelotrichales | Erysipelotrichaceae | Group ZOR0006 |
| OTU-063 | Firmicutes | Bacilli | Lactobacillales | Streptococcaceae | *Streptococcus* |
| OTU-066 | Proteobacteria | Alphaproteobacteria | Rhodobacterales | Rhodobacteraceae | Unclassified |
| OTU-070 | Actinobacteriota | Actinobacteria | Corynebacteriales | Corynebacteriaceae | *Corynebacterium* |
| OTU-071 | Actinobacteriota | Actinobacteria | Corynebacteriales | Corynebacteriaceae | *Corynebacterium* |
| OTU-072 | Proteobacteria | Gammaproteobacteria | Enterobacterales | Pseudoalteromonadaceae | *Pseudoalteromonas* |
| OTU-073 | Verrucomicrobiota | Lentisphaeria | Victivallales | Victivallaceae | Unclassified |
| OTU-074 | Proteobacteria | Gammaproteobacteria | Enterobacterales | Vibrionaceae | *Photobacterium* |
| OTU-075 | Firmicutes | Clostridia | Lachnospirales | Lachnospiraceae | *Blautia* |
| OTU-077 | Proteobacteria | Gammaproteobacteria | Enterobacterales | Pseudoalteromonadaceae | *Pseudoalteromonas* |
| OTU-078 | Bacteroidota | Bacteroidia | Flavobacteriales | Flavobacteriaceae | *Tenacibaculum* |
| OTU-079 | Bacteroidota | Bacteroidia | Cytophagales | Hymenobacteraceae | *Adhaeribacter* |
| OTU-080 | Proteobacteria | Alphaproteobacteria | Sphingomonadales | Sphingomonadaceae | *Erythrobacter* |
| OTU-081 | Firmicutes | Clostridia | Clostridiales | Clostridiaceae | *Clostridium* sensu stricto 1 |
| OTU-082 | Actinobacteriota | Actinobacteria | Corynebacteriales | Nocardiaceae | *Nocardia* |
| OTU-089 | Proteobacteria | Alphaproteobacteria | Defluviicoccales | Defluviicoccaceae | *Defluviicoccus* |
| OTU-095 | Actinobacteriota | Actinobacteria | Micrococcales | Micrococcaceae | *Rothia* |
| OTU-100 | Cyanobacteria | Cyanobacteriia | Cyanobacteriales | Chroococcidiopsaceae | *Chroococcidiopsis* |
| OTU-105 | Proteobacteria | Gammaproteobacteria | Salinisphaerales | Salinisphaeraceae | *Salinisphaera* |
| OTU-107 | Firmicutes | Clostridia | Peptostreptococcales-Tissierellales | Family_XI | *Anaerococcus* |
| OTU-111 | Firmicutes | Bacilli | Lactobacillales | Streptococcaceae | *Streptococcus* |
| OTU-112 | Firmicutes | Bacilli | Staphylococcales | Gemellaceae | *Gemella* |
| OTU-113 | Actinobacteriota | Actinobacteria | Corynebacteriales | Corynebacteriaceae | *Corynebacterium* |
| OTU-121 | Firmicutes | Bacilli | Mycoplasmatales | Mycoplasmataceae | *Mycoplasma* |
| OTU-122 | Patescibacteria | Parcubacteria | *Candidatus* Nomurabacteria | *Candidatus* Nomurabacteria | Unclassified |
| OTU-123 | Proteobacteria | Alphaproteobacteria | Rickettsiales | *Candidatus* Hepatincola | Unclassified |
| OTU-141 | Proteobacteria | Gammaproteobacteria | Enterobacterales | Vibrionaceae | *Vibrio* |

**Table S4**. PERMANOVA of the bacterial operational taxonomic units (OTU) richness in the midgut of 12 fish species from the Aegean Sea. Greece. Red characters indicates p < 0.05.

|  | ***Diplodus annularis*** | ***Diplodus vulgaris*** | ***Euthynnus alleteratus*** | ***Mullus surmuletus*** | ***Pagrus pagrus*** | ***Pagellus erythrinus*** | ***Spondyliosoma cantharus*** | ***Scyliorhinus canicula*** | ***Sparisoma cretense*** | ***Scorpaena porcus*** | ***Scorpaena scrofa*** | ***Uranoscopus scaber*** |
| --- | --- | --- | --- | --- | --- | --- | --- | --- | --- | --- | --- | --- |
| ***Diplodus annularis*** |  | 0.366 | 0.063 | 0.889 | 0.293 | 0.176 | 0.200 | 0.113 | 0.829 | 0.369 | 0.249 | 0.252 |
| ***Diplodus vulgaris*** |  |  | 0.029 | 0.286 | 0.767 | 0.513 | 0.602 | 0.547 | 0.167 | 0.055 | 0.403 | 0.917 |
| ***Euthynnus alleteratus*** |  |  |  | 0.208 | 0.030 | 0.029 | 0.030 | 0.029 | 0.029 | 0.059 | 0.027 | 0.098 |
| ***Mullus surmuletus*** |  |  |  |  | 0.175 | 0.114 | 0.145 | 0.145 | 0.620 | 0.486 | 0.121 | 0.304 |
| ***Pagrus pagrus*** |  |  |  |  |  | 0.482 | 0.571 | 0.578 | 0.111 | 0.030 | 0.397 | 0.970 |
| ***Pagellus erythrinus*** |  |  |  |  |  |  | 0.947 | 0.971 | 0.087 | 0.030 | 0.913 | 0.630 |
| ***Spondyliosoma cantharus*** |  |  |  |  |  |  |  | 0.913 | 0.090 | 0.029 | 0.972 | 0.602 |
| ***Scyliorhinus canicula*** |  |  |  |  |  |  |  |  | 0.084 | 0.029 | 1.000 | 0.628 |
| ***Sparisoma cretense*** |  |  |  |  |  |  |  |  |  | 0.146 | 0.054 | 0.197 |
| ***Scorpaena porcus*** |  |  |  |  |  |  |  |  |  |  | 0.028 | 0.055 |
| ***Scorpaena scrofa*** |  |  |  |  |  |  |  |  |  |  |  | 0.597 |
| ***Uranoscopus scaber*** |  |  |  |  |  |  |  |  |  |  |  |  |

**Table S5**. PERMANOVA of the bacterial operational taxonomic units (OTU) abundances in the midgut of 12 fish species from the Aegean Sea. Greece. Red characters indicate p < 0.05. Shaded cells indicate trophic habit: Red: omnivore with a preference for animal material; Green: carnivore with a preference for fish and cephalopods; Blue: carnivore with a preference for decapods and fish; Yellow: omnivore with a preference for plants.

|  | *Diplodus*  *annularis* | *Diplodus*  *vulgaris* | *Euthynnus*  *alletteratus* | *Mullus*  *surmuletus* | *Pagrus*  *pagrus* | *Pagellus*  *erythrinus* | *Spondyliosoma*  *cantharus* | *Scyliorhinus*  *canicula* | *Sparisoma*  *cretense* | *Scorpaena*  *porcus* | *Scorpaena*  *scrofa* | *Uranoscopus*  *scaber* |
| --- | --- | --- | --- | --- | --- | --- | --- | --- | --- | --- | --- | --- |
| *Diplodus*  *annularis* | - | 0.085 | 0.114 | 0.026 | 0.031 | 0.029 | 0.059 | 0.200 | 0.030 | 0.056 | 0.114 | 0.5469 |
| *Diplodus*  *vulgaris* |  | - | 0.090 | 0.581 | 0.174 | 0.110 | 0.310 | 0.419 | 0.600 | 0.326 | 0.457 | 0.9156 |
| *Euthynnus*  *alletteratus* |  |  | - | 0.057 | 0.030 | 0.057 | 0.459 | 0.408 | 0.028 | 0.027 | 0.264 | 0.3239 |
| *Mullus*  *surmuletus* |  |  |  | - | 0.207 | 0.143 | 0.087 | 0.057 | 0.147 | 0.060 | 0.170 | 0.369 |
| *Pagrus*  *pagrus* |  |  |  |  | - | 0.403 | 0.028 | 0.118 | 0.111 | 0.090 | 0.112 | 0.2027 |
| *Pagellus*  *erythrinus* |  |  |  |  |  | - | 0.027 | 0.087 | 0.058 | 0.058 | 0.108 | 0.1946 |
| *Spondyliosoma*  *cantharus* |  |  |  |  |  |  | - | 0.600 | 0.029 | 0.087 | 0.485 | 0.5063 |
| *Scyliorhinus*  *canicula* |  |  |  |  |  |  |  | - | 0.085 | 0.145 | 0.484 | 0.8893 |
| *Sparisoma*  *cretense* |  |  |  |  |  |  |  |  | - | 0.058 | 0.091 | 0.3166 |
| *Scorpaena*  *porcus* |  |  |  |  |  |  |  |  |  | - | 0.144 | 0.2106 |
| *Scorpaena*  *scrofa* |  |  |  |  |  |  |  |  |  |  | - | 0.6388 |
| *Uranoscopus*  *scaber* |  |  |  |  |  |  |  |  |  |  |  | - |

**Table S7**. Range and median values of the coefficient of variation, i.e., the ratio of the standard deviation to the average, of the most dominant OTUs and the number of shared between the individuals found in each fish species.

|  | **Coefficient of variation** | | |  |
| --- | --- | --- | --- | --- |
|  | **Minimum** | **Median** | **Maximum** | **Shared OTUs** |
| All 12 species | 126.1% | 496.4% | 666.0% | 61 (7.1%) |
| *Diplodus annularis* | 0.0% | 81.6% | 200.0% | 55 (12.6%) |
| *Diplodus vulgaris* | 0.0% | 116.1% | 200.0% | 38 (9.0%) |
| *Euthynnus alletteratus* | 16.3% | 93.6% | 200.0% | 36 (12.8%) |
| *Mullus surmuletus* | 10.3% | 113.5% | 200.0% | 29 (11.6%) |
| *Pagrus pagrus* | 0.0% | 50.8% | 173.2% | 45 (25.1%) |
| *Pagellus erythrinus* | 0.0% | 66.9% | 173.2% | 39 (21.0%) |
| *Spondyliosoma cantharus* | 8.1% | 68.8% | 200.0% | 34 (15.8%) |
| *Scyliorhinus canicula* | 8.4% | 100.9% | 200.0% | 45 (18.3%) |
| *Sparisoma cretense* | 15.2% | 91.3% | 200.0% | 27 (13.0%) |
| *Scorpaena porcus* | 10.5% | 115.5% | 200.0% | 42 (15.9%) |
| *Scorpaena scrofa* | 12.8% | 99.1% | 200.0% | 37 (15.4%) |
| *Uranoscopus scaber* | 24.7% | 117.4% | 173.2% | 34 (15.2%) |


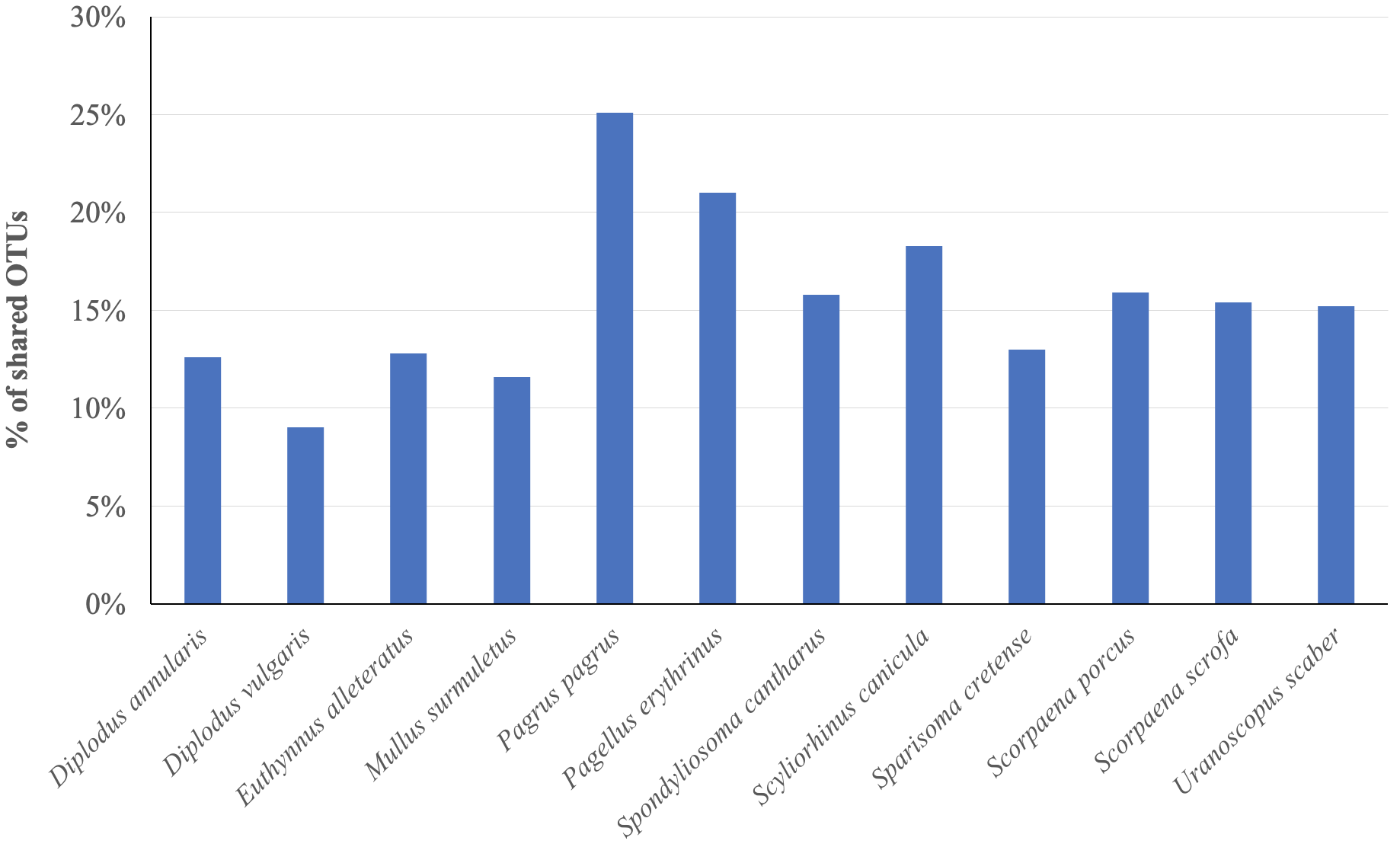


**Figure S1**. Shared operational taxonomic units in the midgut bacterial communities among the individuals of each of the 12 fish species from the Aegean Sea.


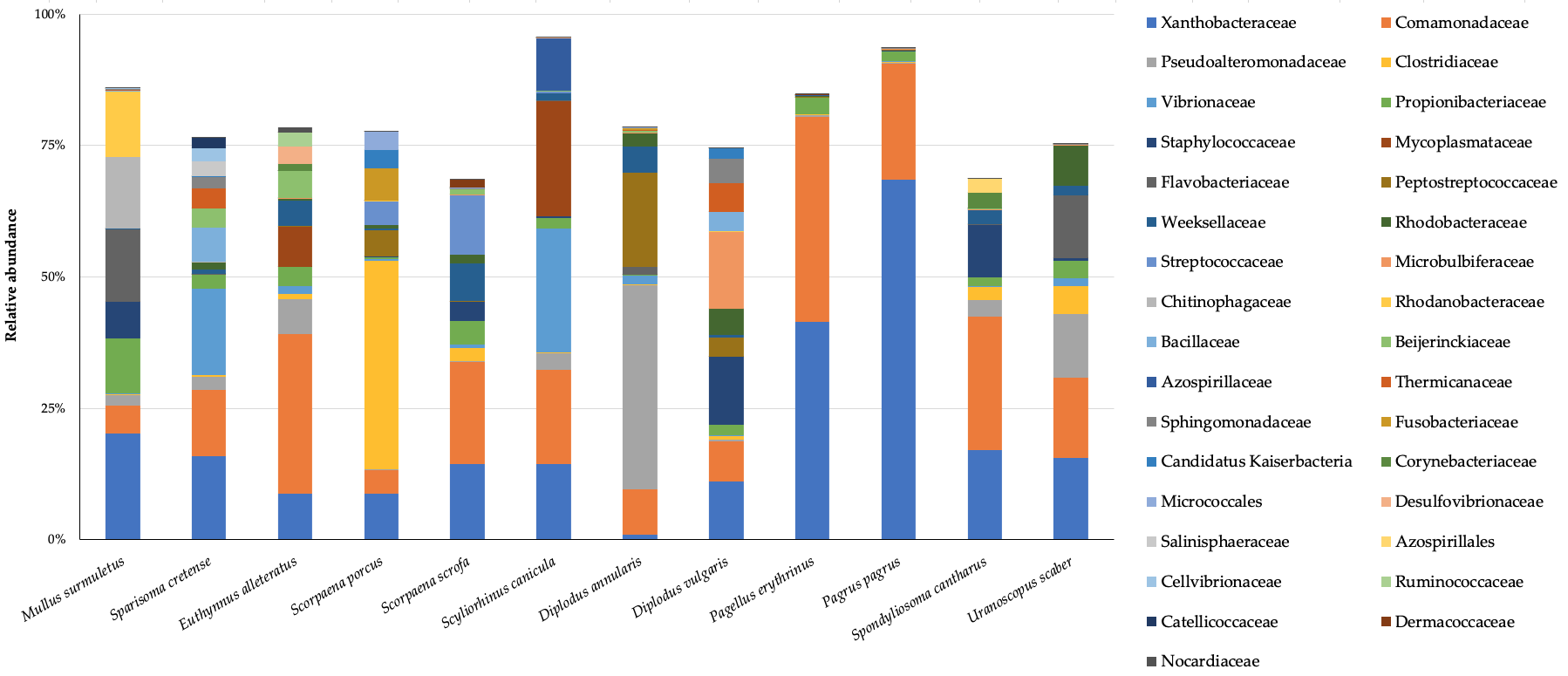


**Figure S2a**. Taxonomic composition (31 families and two orders) of the most dominant (≥70% cumulative relative abundance) bacterial operational taxonomic units of the 12 fish species. Taxa in the legend are shown in decreasing total abundance in the whole dataset.


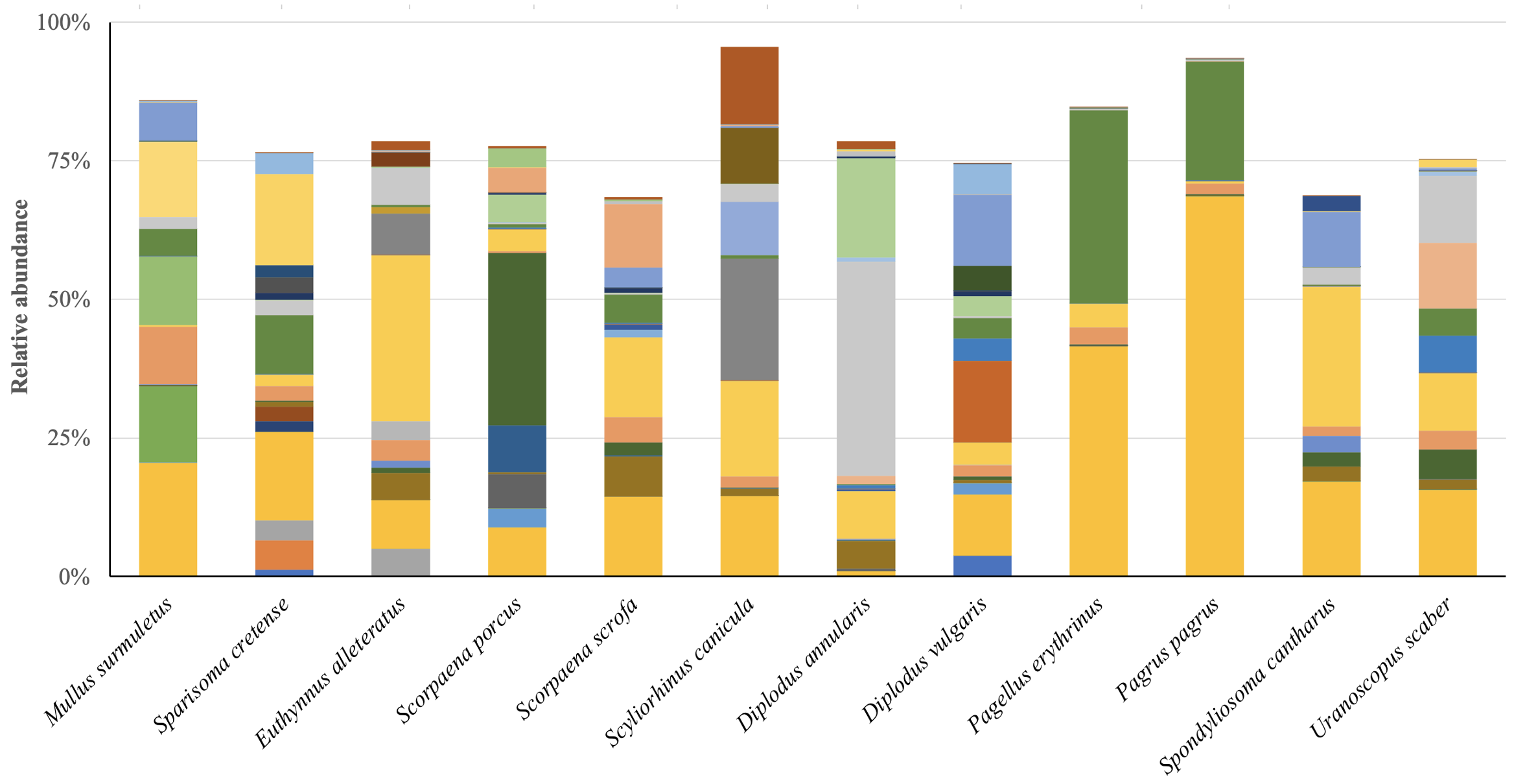


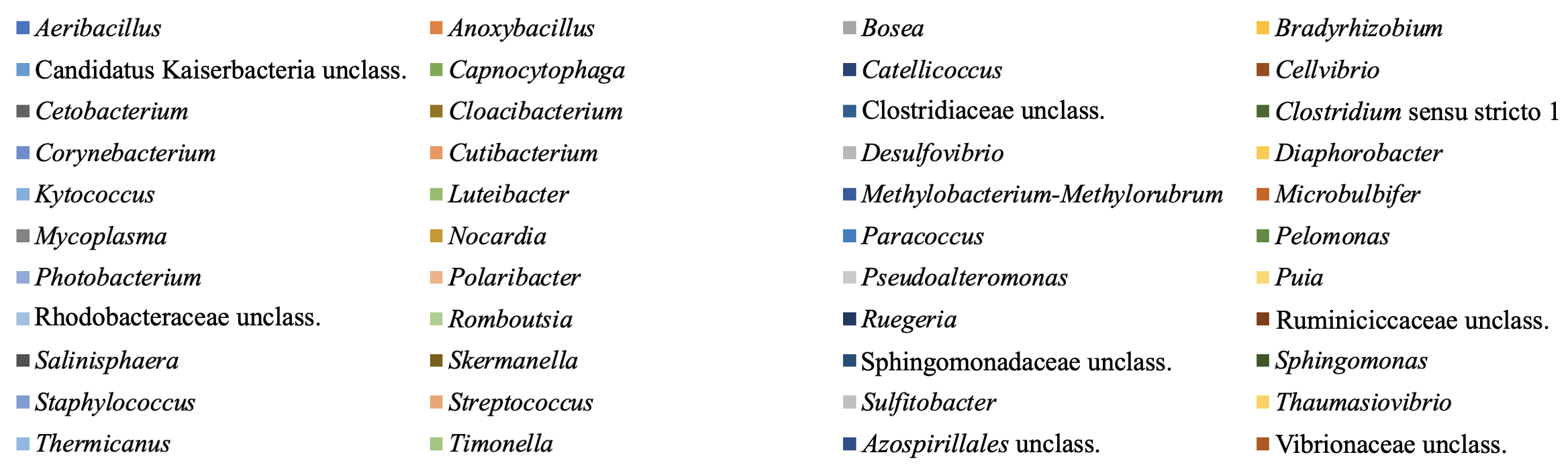


**Figure S2b**. Taxonomic composition at the genus level or higher of the most dominant (≥70% cumulative relative abundance) bacterial operational taxonomic units of the 12 fish species. Note: multiple OTUs belonging to the same genus or higher taxon were pooled together.


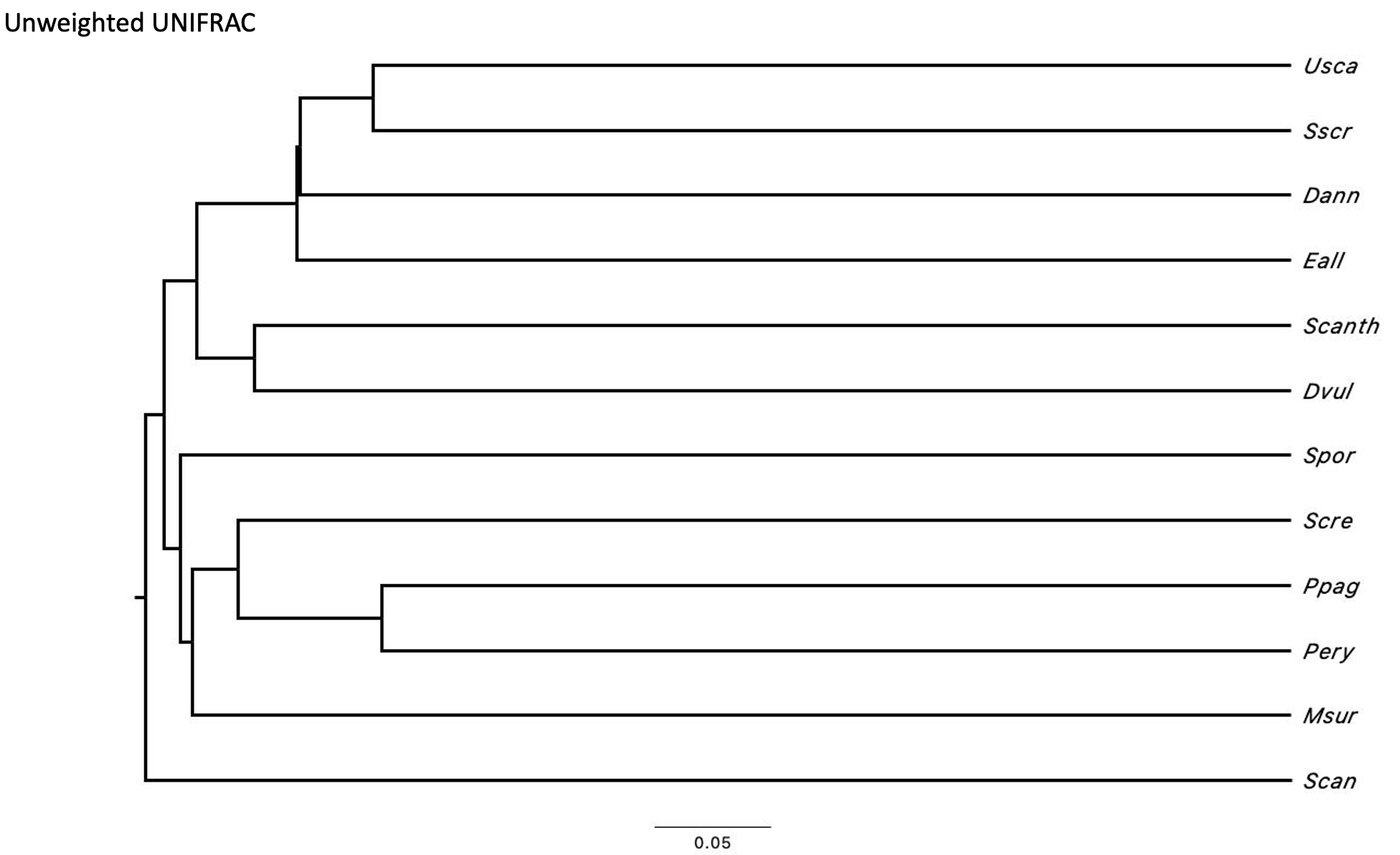


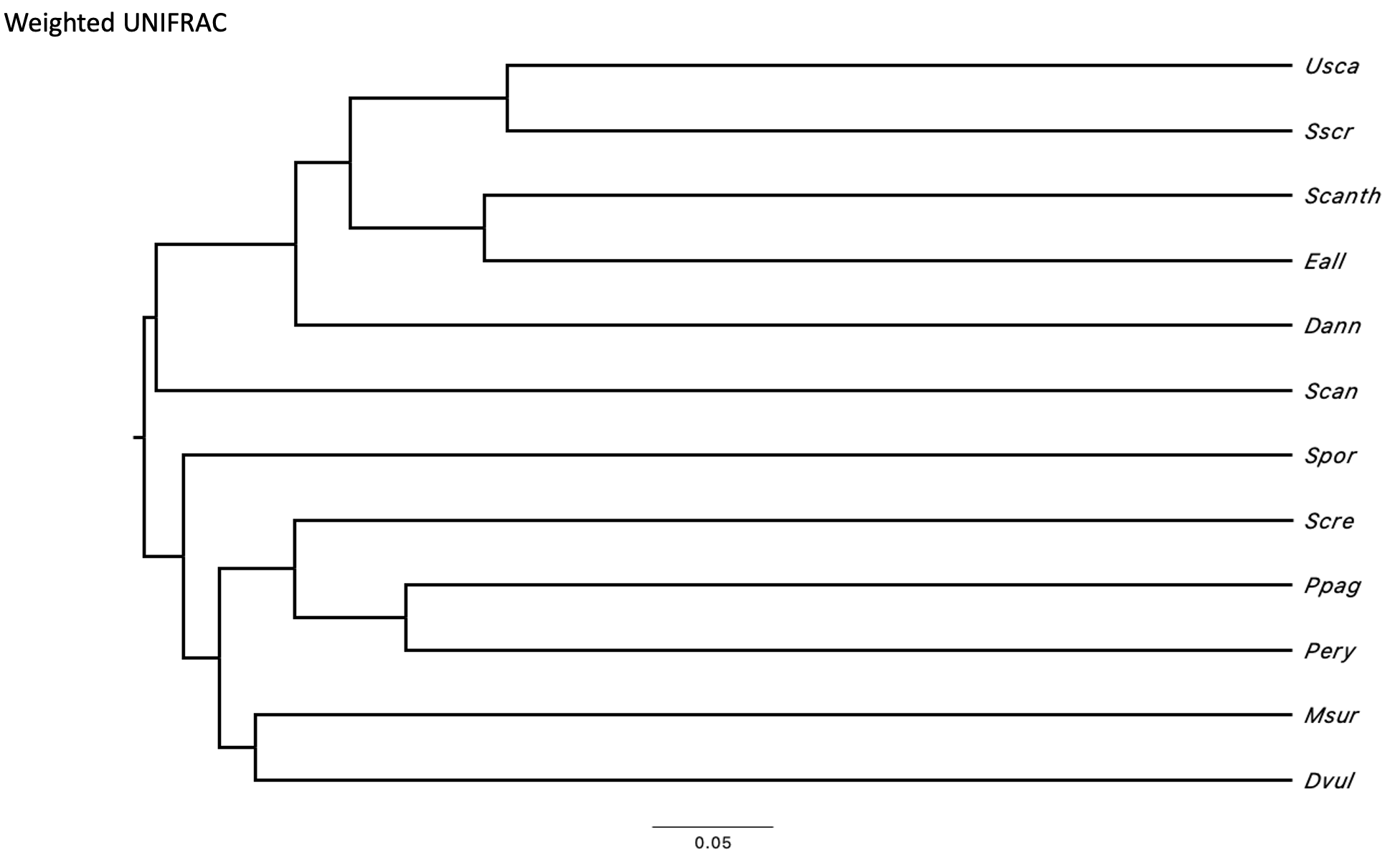


**Figure S3.** Unweighted and weighted UniFrac analyses of the 12 fish species’ midgut bacterial microbiota.


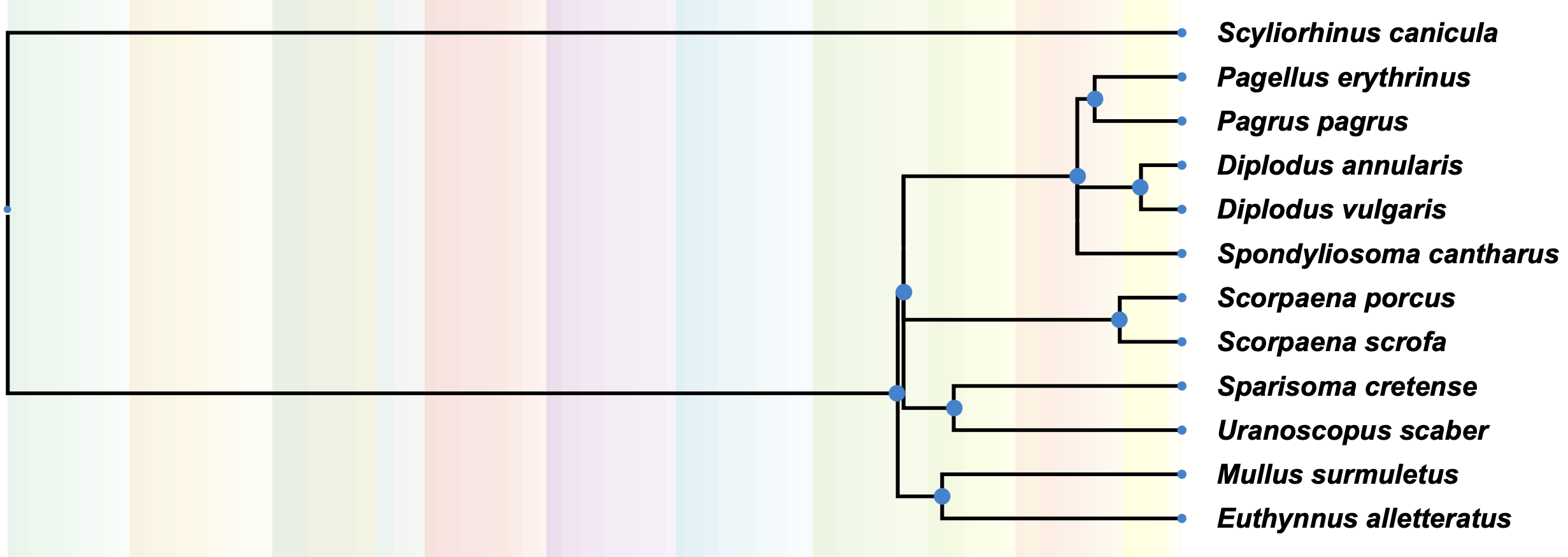


**Figure S4**. The phylogenetic relations of 11 of the 12 fish species from the Aegean Sea according to <http://www.timetree.org>.
